# Psycholytic Therapy Using LSD: a Realist Review

**DOI:** 10.1002/brb3.71187

**Published:** 2026-04-15

**Authors:** Hamish Grime, Eugenia Drini

**Affiliations:** ^1^ University College London London UK; ^2^ University College London, Goldsmiths University of London London UK

**Keywords:** LSD, mental health, psychedelic, psycholytic therapy

## Abstract

**Purpose:**

Psycholytic therapy, utilizing psychoactive compounds such as d‐lysergic acid diethylamide (LSD) to enhance psychoanalytically oriented therapy, was once the most common form of psychedelic‐assisted psychotherapy. Despite its historical prominence, little attention has been given to the reasons behind its decline, and there is a lack of standardized guidance for new clinical trials. This review addresses the diversity of psycholytic methods and their association with effectiveness and safety.

**Method:**

A realist review was conducted in which studies were identified via a comprehensive bibliography of publications on psycholytic therapy with entheogens from 1948 to 1995. Intervention variables were examined in relation to rates of adverse events and diagnosis‐adjusted improvement, allowing for an analysis of risk factors and practices associated with positive outcomes.

**Findings:**

Analysis of studies published from 1954 to 1965 revealed several features conducive to successful therapy: using a flexible, intuitive therapeutic approach; promptly correcting transference issues; maintaining therapist presence throughout; providing more than ten treatment sessions and extensive preparatory work; incorporating creative activities during integration; using maximal tolerated doses; and avoiding abrupt pharmacological termination.

**Conclusions:**

Psycholytic therapy has shown potential to significantly improve severe mental health conditions, especially when trust in the therapist is fostered. Developing a strong therapeutic relationship helps address trauma‐based responses resistant to psychotherapy alone, while rapid resolution of internal conflicts may ease the subsequent integration process.

## Graphical abstract

Psycholytic therapy using d‐lysergic acid diethylamide (LSD) appears safe and therapeutically effective when delivered with strong therapeutic trust, extensive preparation, and more than ten sessions. Flexible, relationally focused methods and creative integration work are associated with better outcomes and reduced adverse events.

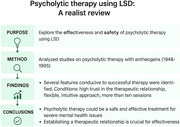

## 1 Introduction

### 1.1 LSD

Albert Hofmann was the first on record to ingest d‐lysergic acid diethylamide (LSD) in 1943 (Hofmann 2022, 10). As a consequence of its widespread use both inside and outside of psychiatry, it became a prototype for this elusive group of compounds (Grinspoon and Bakalar 1979), sometimes known as entheogens (Ruck et al. 1979). LSD research is less than a century old, and for some decades following the United Nations Convention on Psychotropic Substances (1971) it was stifled under regulation (Bewley‐Taylor, 2003).

A critical therapeutic mechanism with which entheogens have been credited is “ego‐dissolution” (Nour et al. 2016), from which mystical experiences can emerge (Pahnke 1969; Roseman et al. 2018). The apparent directness, objectivity, and reproducibility of this effect closely resemble other psychiatric medications (Gearin and Devenot 2021), the primary category into which it has been inaugurated. However, warnings against such a conception have been issued from within this domain: “We cannot emphasize too strongly, however, that the drug [LSD] does not fall into the group of “physical” treatments and that it should be used only by experienced psychotherapists and their assistants.” (Sandison et al. 1954). To illustrate the cultural determinants of their conceptualization, entheogens are elsewhere conceived of as ancestors, spirits, or plant‐helper allies who empower the participant to explore myths and cosmologies (Gearin and Devenot 2021). Research trials do sometimes evoke religion in the setting (Golden et al. 2022; Pollan 2019, 55), and the therapeutic value of mystical experiences has been affirmed many times (Ko et al. 2022). Despite this, one of the most common scales operationalizing mystical experience contains the factor of ineffability (Barrett et al. 2015: Mystical Experience Questionnaire), therein marginalizing alternative philosophies. In the U.K., only neuroscientific studies are permitted the use of compounds that do not conform to Good Manufacturing Practice standards (Nutt et al. 2013), and similarly medicalized findings have emerged from this field.

The intensity of responses to given doses of LSD are not reproducible. Dose‐response relationships vary widely between individuals (Sandison et al. 1954), but do not depend on weight (Agnew and Hoffer 1955). Furthermore, an individual receiving a repeated dose at a different time may produce responses of different intensity (Sandison et al. 1954). The effects may last 4 to 8 h but may occasionally extend to ten days (Sandison et al. 1954).

### 1.2 Psycholytic Therapy

The treatment approach designed to produce a single overwhelming mystical religious peak experience may be called psychedelic (Yensen, 1985). The alternative psycholytic approach uses entheogens to facilitate ongoing psychoanalytically oriented therapy (Leuner 1967). They have previously been distinguished by dose alone (Batievsky et al. 2023), but in the absence of standardised procedures (Passie 2024), the typical features are inconsistent (Passie 2024) and may overlap, especially when individually tailored. Nevertheless, the psycholytic patient should remain in communication with their attendant, and recognize themselves as participating in therapy (Passie 1997).

Psycholytic therapy benefits from a continuity of theoretical and applied developments from within the culture where it originated. In the 1920s and 1930s, psychoanalysts would sometimes use baribiturates to enhance patient recall and disclosure in a treatment approach known as “narcoanalysis” (Passie 1997). The first English language research publication on LSD presented it as a tool for expediting psychotherapy (Busch and Johnson, 1950, as cited in Carhart‐Harris and Goodwin 2017). Since then, research has demonstrated LSD's potential to intensify transference (Cutner 1959), facilitate access to early memories (Soskin 1973), induce regression to associated experiences (Bierer and Browne 1960), and enhance free association by promoting primary process thinking (Guss 2022). Stan Grof—the late expert in LSD therapies—asserted that observations from these treatments could be considered laboratory proof of the Freudian theoretical framework if they were the only type of experience produced (1980, 64). Various psychoanalytically‐oriented frameworks could be harnessed to explain and address the biographical experiences central to psycholytic therapy (Leuner 1967).

### 1.3 The Scope of Psycholytic Therapy

The active sessions in psycholytic therapy need not be identical to any conventional psychotherapy. Entheogenic experiences can merely be used to support primarily conventional psychoanalytical treatments in the intervening periods (Passie 1997). Hanscarl Leuner (Passie et al. 2022, 2), who founded the European Medical Society for Psycholytic Therapy, developed the technique of Guided Affective Imagery (Passie 2024). Specific scenes would be roughly described, such as a house, the view from a mountain, or a relation. The analyst would encourage the patient to describe sensory details, emotional features, and story developments (Leuner 1969). Ronald Sandison, who coined the word “psycholytic” in 1960 (Leuner, 1967) and became the first psychiatrist on record to bring LSD to the United Kingdom (Roberts, 2008, 44), incorporated Jungian psychology and art therapy (Roberts, 2008, 46).

Psychotherapies unrelated to psychoanalysis may also belong under the umbrella of psycholytic therapy, but the standardized form this eventually takes will inevitably be distinguished from the above examples as procedures such as dose and the number of intervening sessions are tailored accordingly. Though its original definition referred only to LSD (Leuner 1967), where the strongest evidence base remains, the most extensive bibliography of psycholytic therapy encompasses any entheogen (Passie 1997).

Psycholytic research has been neglected in recent years (Passie, 2022), obscuring the scope of methods already explored. The variability in treatments creates uncertainty for researchers planning to launch a pilot study, and direct comparisons between methods remain rare.

### 1.4 Aims and Objectives

The aim of this research is to lay the groundwork for the first English‐language pilot study of psycholytic therapy in this century. The objectives are three: first, to orient researchers toward procedural variables that have already been studied and reported on in English; second, to suggest how differences in effectiveness are brought about by changes in these variables; and finally, to identify indicators of risk in psycholytic therapy.

## 2 Methods

Outcomes in entheogenic therapy are critically sensitive to the quality and quantity of preparatory sessions and integration sessions, as well as the therapeutic context accompanying administration (Hofmann 2022, 74; Sandison et al. 1954). We conducted a realist review because it is designed with the flexibility to account for complex interventions by developing and iteratively refining an initial program theory (Pawson et al. 2004).

This realist review follows the procedure suggested by Pawson et al. (2005). The reporting adheres to the Realist and Meta‐narrative Evidence Synthesis project guidelines (Wong et al. 2013). In this section, we delineate the source of our data, the eligibility criteria, and the analytical procedure.

### 2.1 Defining the Scope of Research

We initially incorporated all forms of psycholytic therapy with any entheogen, but during preliminary searches we discovered feasible opportunities to narrow this scope in line with our objectives. Realizing that the vast majority of psycholytic therapy used LSD in individual psychotherapy, we focused exclusively on research with this entheogen and excluded group therapy. Distinguishing between LSD and psilocybin is possible for some participants (Leuner 1967), but not all (Abramson and Rolo 1967). A controlled trial investigating their somatic, perceptual, affective, and cognitive effects demonstrated remarkable consistency between the two (Hollister and Hartman 1962). Group psycholytic therapy is unlikely to be used in initial psycholytic trials because therapists in such a situation have reported challenges orchestrating group communication (Hausner and Doležal 1963).

It became evident that most studies published before the year approximately 1980 reported results in terms of the number of participants who improved. Selecting for this reportage supported the comparability of outcome data.

Finally, most extant studies reported their results by diagnosis, which presented the opportunity to control for this variable. Therefore, the scope was narrowed to such studies.

### 2.2 Searching

Initial searches did not uncover any examples of psycholytic therapy conducted after 1995 that were reported in English. Therefore, potentially eligible studies were first identified from a chapter of a bibliography of all psycholytic studies with any entheogen published between 1948 and 1995 (Passie 1997). Full texts were searched for on Web of Science, Ovid, PsychINFO, and PubMed. If unsuccessful, we searched Google Scholar and physical libraries. Final attempts were made on the first two pages of Google.

### 2.3 Study Selection

Data from all eligible individuals were used to develop and refine the program theory. Ineligible studies were inspected to determine if any individuals met the criteria, who would then be included.

#### 2.3.1 Inclusion Criteria

LSD administered.

Trials or case series.

Outcomes stratified by diagnosis.

Reported in the English language.

#### 2.3.2 Exclusion Criteria

Group therapy.

Participants with multiple diagnoses, obsolete sexual neuroses, or diagnoses too rare to make comparisons (Appendix A).

### 2.4 Critical Appraisal

The authors of the included studies rarely attempted to control any variables, instead attempting to mobilize the entire context to bring about the desired outcome. With the aim of accounting for all relevant data in the program theory, realist reviews reject formal appraisal of study quality (Popay, 2006, 2). Study rigor was indicated by a custom appraisal table and the reliability of the outcome. Study relevancy was indicated by the transparency of the intervention.

### 2.5 Data Extraction and Analysis

We developed and refined our program theory by analyzing the interventions in relation to improvements and adverse events. Preliminary analyses of these components are described below.

#### 2.5.1 Intervention

We created a data extraction tool to simplify the interventions for comparison, which documented the following: author, publishing date, mean number of psycholytic sessions, upper range of usual doses, dosing strategy, presence of preparatory sessions, attitude of clinician, transference or abreactive orientation, persistence of clinician attendance, and session termination by other pharmacological means. These factors reflected deliberate attempts by the study authors to highlight adaptations that might benefit or harm their patients.

#### 2.5.2 Outcome Data

In order to adjudicate between interventions according to their outcomes, we compared studies according to the number of patients reported to have made certain levels of improvements. Based on guidance for systematic reviews, we allocated a common rubric to the most precise scoring system in order to maintain statistical power (Popay et al. 2006). The common rubric was the most precise scale of included studies (Chandler and Hartman 1960). Possible outcomes were little or no change (0), slight improvement (1), some improvement (2), considerable improvement (3), marked improvement (4), outstanding improvement (5).

The correspondence between different outcome scales were presented in Appendix B. Translating outcomes on less precise scales to the common rubric entailed collapsing them into their medians. In doing so, we assumed that improvements were evenly distributed within outcome categories. Authors of one study indicated that no patients achieved “slight improvements” (Sandison et al. 1954), prompting its removal as a possibility.

#### 2.5.3 Statistical Adjustment

The frequency with which diagnostic information was reported impelled us to adjust the improvement rates by diagnostic data, enhancing reliability despite precluding analysis of which populations could benefit most from the interventions.

First, participants were organized into five categories (Appendix A): Depression; Anxiety; Obsessive‐compulsive disorders; Hysteria; Psychotic disorders; Personality disorders. This was done in accordance with contemporary understandings of prognosis unless the fifth edition of the Diagnostic and Statistical Manual (DSM) did not list an equivalent mental health condition (American Psychiatric Association 2013), in which case the historical diagnostic framework took precedence. The combination of similar but different categories, or “hybrid items,” was based on recommendations for addressing missing data during exploratory synthesis (Yeaton et al. 1995).

## 3 Results

### 3.1 Study Selection

**FIGURE 1:**
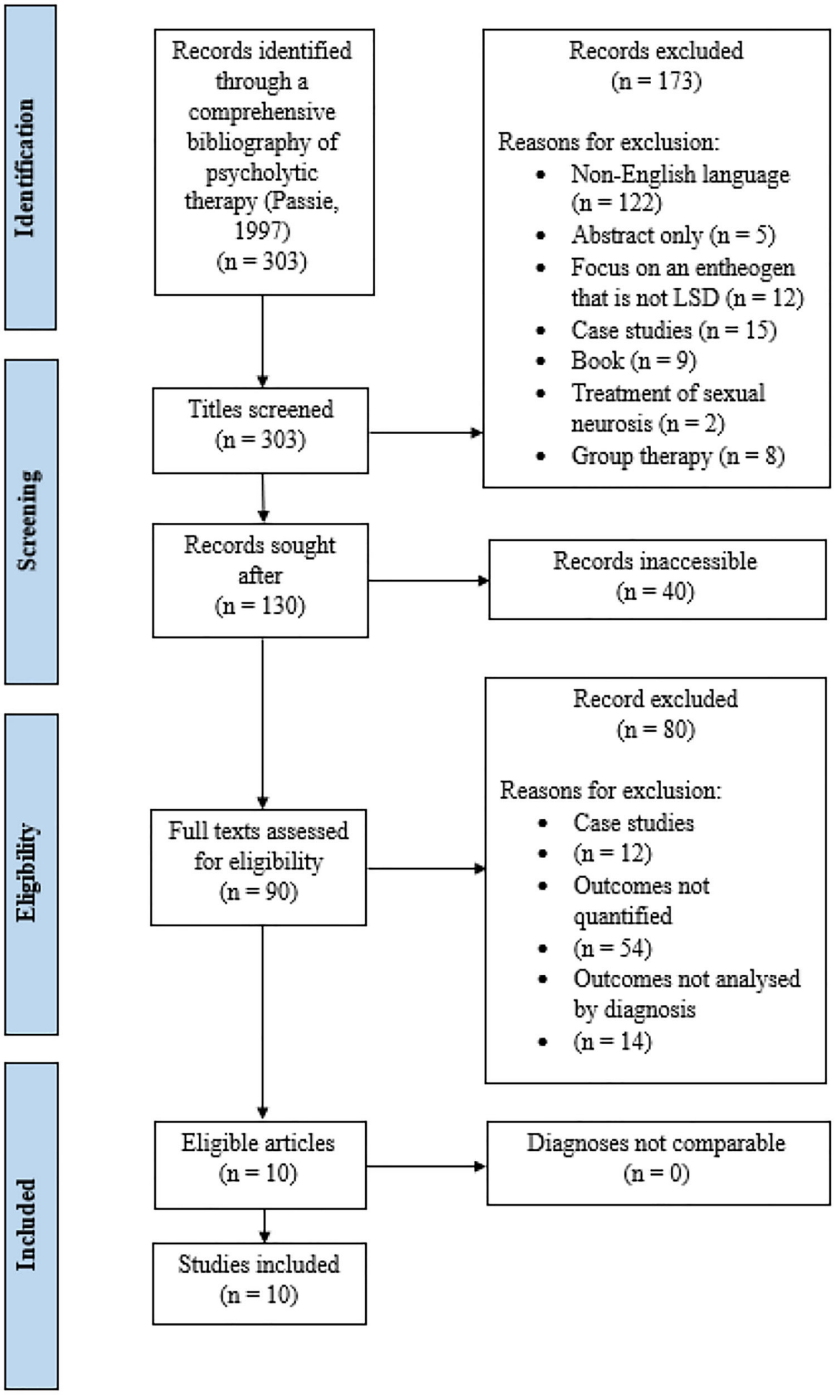
PRISMA diagram of study selection.

Three hundred and three psycholytic research papers were published between 1948 and 1995 (Passie 1997). Of the 181 written in the English language, 40 were inaccessible (Figure 1). Either the locations of the relevant journal editions, books, or congress proceedings were not readily disclosed on the internet, or the expense of purchase and international shipping was too great to justify their potential inclusion. Ten research papers satisfied all eligibility criteria.

### 3.2 Study Characteristics

Authors of eligible trials were reporting on their work in the United Kingdom, the United States of America, Germany, Denmark, and Australia between 1954 and 1967 inclusive. Two of these authors have been extolled as “pioneers,” namely Ronald Sandison (Grof 1980, 22) and Hanscarl Leuner (Passie et al. 2022, 2).

Two studies reported results by individual (Eisner and Cohen 1958; Martin 1957); the rest by diagnosis. Sandison and Whitelaw (1957) published two different methods with corresponding results in the same paper. The main trial (Sandison and Whitelaw, 1957a) is cited separately from the specialized treatment for people with schizophrenia (Sandison and Whitelaw, 1957b).

**FIGURE 2:**
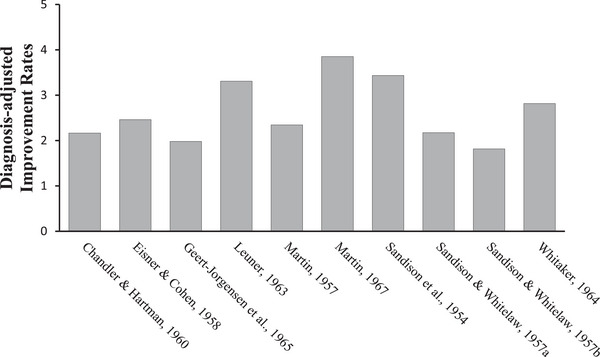
Diagnosis‐adjusted improvement rates by study.

Five adverse events were reported: three suicides, a homicide, and a brief psychotic episode. Diagnosis‐adjusted improvement rates ranged from moderate improvements (1.78; Sandison and Whitelaw, 1957b) to considerable improvements (3.78; Martin 1967). These are illustrated below (Figure 2) and enumerated by study and by diagnosis in Appendix C. All participants were invariably described as treatment resistant, except for 63% of a single sample (Leuner 1963). All studies that detailed positive selection criteria cited ego strength or motivation for recovery (Eisner and Cohen 1958; Geert‐Jörgensen et al. 1964; Leuner 1963; Martin 1957; Sandison and Whitelaw, 1957a; Whitaker 1964).

### 3.3 Main Findings

The main findings concern the effects of eight independent variables (Appendix D) implicated in the safety or effectiveness of psycholytic therapy. The information for the methods of Leuner (1963) was filled in primarily from Passie (2022), who worked alongside Leuner.

#### 3.3.1 Number of Sessions

There were usually between 4 and 10 active sessions of psycholytic therapy provided (Eisner and Cohen 1958; Leuner 1963; Sandison et al. 1954; Sandison and Whitelaw, 1957a; Sandison and Whitelaw, 1957b). Trials with means of 20 (Martin 1967) and 33 sessions (Whitaker 1964) were associated with greater improvement rates. These were provided weekly (Martin 1967; Sandison and Whitelaw, 1957a; Sandison and Whitelaw, 1957b), with some individual variation reported based on the volume and nature of material uncovered (Chandler and Hartman 1960; Eisner and Cohen 1958).

#### 3.3.2 Dosing

Participants were typically administered with around 100–150 µg of LSD. Whitaker (1964) reported an upper limit for usual doses of 250 µg. Usual doses of up to 400 µg occurred in two studies (Sandison et al. 1954; Sandison and Whitelaw, 1957a), one of which reported four participants being talked out of suicide while intoxicated (Sandison and Whitelaw, 1957a). Anomalous doses of up to 1,600 µg were associated with two of the three suicides in this review, the only homicide, and a high dropout rate of 27% (Geert‐Jörgensen et al. 1964).

Some psychiatrists utilized an incremental dosing strategy whereby an initially low dose of 25–50 µg was increased in subsequent sessions by the same amount until an individually tailored optimum was reached (Chandler and Hartman 1960; Eisner and Cohen 1958; Martin 1957; Sandison et al. 1954). One set of authors decreased the dose again after this peak (Chandler and Hartman 1960). They reported the highest dropout rate of 35% and a suicide.

Improvement rates could not be discerned by the doses used, nor by the presence of an incremental dosing strategy.

#### 3.3.3 Consistency of the Therapist's Attitude

A flexible and intuitive style of psychotherapy was usually recommended by included studies, but Chandler and Hartman's (1960) therapeutic strategy stands out in this regard. They aimed to maintain a friendly yet firm attitude irrespective of the patient or their circumstances and reported one suicide and a brief psychotic episode during treatment. Their procedure was otherwise intended to mimic those of Eisner and Cohen (1958), who achieved greater improvements, less attrition, and no suicides, though these small differences could easily be attributed to the null hypothesis given Eisner and Cohen's (1958) initial sample of just 29. Lending stronger support to the benefits of a flexible approach, the most effective study in the present review made use of preparation sessions to determine suitable ways of personalizing their highly flexible psychotherapy (Martin 1967).

#### 3.3.4 Transference

The most flexible studies also utilized an anaclitic approach (Eisner and Cohen 1958; Martin 1967): they placed a strong emphasis on correcting previously traumatic relationships by satisfying the patient's needs in the present (Grof 1980, 38). Therefore, therapists were usually required to play a parental role, using a male and female dyad (Eisner and Cohen 1958) or even providing warm milk (Martin 1967). Martin (1967) had the best improvement rates, and Eisner and Cohen (1958) had improvement rates above the mean of included studies. The latter study had the lowest sample size and lowest outcome precision, so conclusions should weigh heavier on the results of Martin (1967).

Together with Chandler and Hartman (1960), these three studies prioritized the transference relationship over abreaction alone. Here, the discussions and relationship with the therapist were emphasized, but in research prioritizing cathartic abreaction, participants were encouraged to focus inwards, such as on biographical memories or imagery. Leuner (1963) claimed to follow Sandison et al. (1954) in his method, and together they achieved the second and third highest diagnosis‐adjusted improvements. Hence, both orientations can be effective.

#### 3.3.5 Constancy of Clinician Attendance

Some psychiatrists produced great improvements despite only spending a maximum of one (Sandison et al. 1954) or two hours (Leuner 1963) with their patients. However, in at least one of these a nurse was in constant attendance (Sandison et al. 1954), and other patients who were sometimes left alone only moderately improved on average (Martin 1957; Sandison et al. 1957a; Sandison et al., 1957b). In sum, there was a weak association between any staff member being in constant attendance and the improvement of their patients.

#### 3.3.6 Integration

Martin (1967) achieved excellent results despite being the only study to refrain from reviewing psycholytic sessions and conducting intervening interviews.

In two highly effective studies, creative therapies were conducted after the psycholytic session (Leuner 1963; Sandison et al. 1954).

#### 3.3.7 Preparation

There was a strong correlation between the comprehensiveness of preparation and rates of effectiveness. Its absence in Sandison and Whitelaw (1957a; 1957b) is one of the few changes from Sandison et al. (1954) that may have attenuated their results.

#### 3.3.8 Pharmacological Termination of the Experience

Studies that used barbiturates, chlorpromazine, or phenothylamines to cut the effects of LSD short usually had worse improvement rates. Improvement rates at Powick Hospital were higher before this practice was introduced (Sandison et al. 1954) than after (Sandison and Whitelaw 1957). With the discontinuation of pharmacological termination, improvement rates rose from Martin (1957) to Martin (1967).

### 3.4 Study Appraisal

The relevancy of each study is indicated by the extent of missing information in Appendix D, which also indicates the risk of researcher bias. Many of the included studies were carried out in an environment of impending regulations that was present from the early 1960s (Passie 2024), increasing the probability of bias in the sample selection, the measurement of outcome, and the explanation for dropouts (Appendix E). Without indicating systematic error, reliability is also eroded by the small sample sizes of some studies, large follow‐up times, and imprecise outcome measures (Appendix E). There were no reports of blinding at follow‐up.

## 4 Discussion

### 4.1 Summary of Evidence

This realist review aimed to explore methods in psycholytic therapy and provide guidance for initial pilot studies. Despite the high risk of bias, such an endeavor shows promise: The poorest results in this review equated to moderate improvements in a sample composed exclusively of inpatients with treatment‐resistant schizophrenia, most of whom are likely to be entheogen‐naive.

The following discussion presents the program theory as it traces the effectiveness and safety of psycholytic therapy to relevant variables. Subsequently, threats to reliability and validity are examined. Finally, research methods and questions that may advance the broader field of entheogenic research are explored.

#### 4.1.1 Factors Supporting Improvement

Martin (1967) produced the greatest improvement rates without intervening sessions, providing strong evidence that integration can occur during the psycholytic sessions. This is also implied by attempts in two studies to connect developmental experiences to a new frame of reference in the present (Eisner and Cohen 1958; Whitaker 1964). These treatments produced diagnosis‐adjusted improvement rates above the mean, despite one sample having particularly long‐term mental health conditions (Whitaker 1964). Without a connection to the present, repeated abreaction has occurred without benefit, although in these cases the regression was prompted by hypnosis rather than an entheogen (Sandison 1959).

Improvements in Whitaker's (1964) study could also be attributed to an extensive number of psycholytic sessions, because this study stood out on this dimension alongside Martin (1967). In support, Sandison et al. (1954) claim that six to twelve months of psycholytic therapy is necessary in some cases.

Besides the absence of intervening sessions, Martin (1967) differentiated their technique from Martin (1957) by the constant presence of the clinician, an emphasis on transference, and active emotional support. While none of these appear necessary to obtain considerable improvements on average, improvement rates increased dramatically between the two studies, and other research testifies to their benefits. Psycholytic patients have reported it useful if a therapist is in constant attendance (Buckman 1968) and have been known to rely on the therapist's guidance (Holzinger 1962). Without the reassurance of the therapist, patients are prone to become afraid or retraumatized (Cutner 1959). Grof (1980, 152) expresses concern that where a therapist cannot be depended upon in a moment of need, the patient can lose trust in the relationship. He suggests that a lack of trust will lead to attempts to maintain self‐control and avoid facing difficult aspects of their unconscious alone, echoing a minority of recent qualitative accounts of cancer patients undergoing psychedelic therapy with LSD (Gasser et al. 2014).

Perhaps the nurturing role adopted by Martin (1967) and Eisner and Cohen (1958) similarly contributes to an atmosphere of security and commitment. Stan Grof has declared these factors as crucial for recovery (1980, 152), and psychedelic patients show a preference for flexible and humanistic approaches (Watts et al. 2017). If the development of trust is a vital ingredient, it could also explain the strong correlation between extensive preparation sessions and effectiveness. The five studies with the highest improvement rates can be discerned by their incorporation of either anaclitic techniques (Eisner and Cohen 1958; Martin 1967) or more than one preparation session for all participants (Leuner 1963; Sandison et al. 1954; Whitaker 1964).

The program theory suggested that improvements to various mental health conditions can be enhanced by building and maintaining trust in the therapeutic relationship. This appears to be supported by extensive preparation and an intuitive and flexible therapist in constant attendance. During active sessions, the therapist who adopts a parental role in order to correct issues of transference could relieve the pressure on subsequent integration sessions. Harnessing the participant's creativity during integration may also facilitate this process. The benefits of psycholytic therapy seem cumulative.

#### 4.1.2 Risk Factors

The uneven distribution of deaths in the studies in this review communicates the importance of precautionary measures. Deaths were concentrated primarily in the study administering the highest dose of 1600 µg (Geert‐Jörgensen et al. 1964). Investigations of their sample over 22 years later suggest incomplete reporting of adverse events (Larsen 2016). Over one quarter of all the patients treated with LSD in Denmark from 1959 to 1973 successfully claimed compensation for harm inflicted at the Frederiksberg Hospital (Larsen 2016), where most adverse events in this review took place. Two‐thirds of these reported flashbacks, which received no mention in the original paper. This could be attributed to the gradual activation of flashbacks from unresolved unconscious material (Grof 1980, 195) or the original researchers withholding information, but may also reflect victims exaggerating the harm inflicted in order to maximize their compensation.

Although few methodological details were reported, these suicides and the homicide may be attributed to the high doses administered. Eisner and Cohen (1958) had previously asserted an association between high doses of LSD and suicide, and higher doses of any psychoactive compound are associated with increased side effects.

A study where patients were talked out of suicide during active sessions used doses of 400 µg (Sandison and Whitelaw, 1957a), which also exceeds the 200 µg limit typical of psycholytic studies (Leuner, 1967). This raises the possibility that a combination approach sometimes known as psycholytic therapy was used, which utilizes the gradual breakdown of defenses in psycholytic therapy to later facilitate the emergence of a mystical experience (Yensen 1985). However, it is also possible that the patients’ identities remained intact. A case study involving 1,500 µg failed to produce any unusual external manifestations or self‐reported experiential changes (Grof 1976, 31); only after 38 high‐dose sessions did this participant regress into childhood. Since incremental dosing strategies were used in the two aforementioned studies, high doses may only have been prompted by non‐response feedback. Observable responses, then, may be decoupled from the risk of suicide. The program theory emphasizes setting an evidence‐based maximum dose in advance of treatment, which may be somewhere around 400 µg. Extrapolating from Abramson and Rolo (1967), this is estimated to be approximately equivalent to 70 mg of psilocybin.

The patient of Chandler and Hartman (1960) who died by suicide had made three previous attempts and had again disclosed this intention to the therapist at the outset of treatment. She had received 50 electroshock treatments for depression, addiction, and schizophrenia. While the failure of a single psycholytic session to relieve her suffering may have consolidated her despair, this suicide cannot be meaningfully attributed to the form of treatment.

### 4.2 Limitations

#### 4.2.1 Missing Data

Missing data reduces the reliability of the analysis and increases the risk of bias. The factors that emerged from the synthesis were not reported in all studies, primarily being absent in two (Geert‐Jörgensen et al. 1964; Leuner 1963). Other potentially clinically relevant factors were rarely mentioned, if at all, such as the training and personality of the staff, including their experience with LSD; the frequency of hospital parole; the daily life of the patients; and the patients’ emotional condition and current life situation.

Most studies failed to report the follow‐up time, which was as much as three years in one case (Geert‐Jörgensen et al. 1964). Although psychedelic studies have demonstrated a drop in recovery rates after six months (Carhart‐Harris et al. 2017), the general consensus in the 1960s was, apparently, that psycholytic therapy would not bear its fruit until three to six months after the treatment is completed (Leuner 1967). Thus, the effect of follow‐up times on improvement rates is unknown.

#### 4.2.2 Diagnostic Groupings

Adjusting by diagnosis increased the internal validity of comparisons between heterogeneous samples but compromised reliability. The diagnoses that were sorted into the same group may not all be associated with similar prognoses. For example, the “psychotic disorders” group included many participants with schizophrenia, but from one study only participants with paranoia that did not qualify for schizophrenia were assigned to this group (Geert‐Jörgensen et al. 1964), inflating their diagnosis‐adjusted improvement rates. Our retrospective grouping took place up to 67 years after the original studies were published, during which time the diagnostic frameworks have undergone substantial development. To the extent that this is culturally invalid, reliability is further undermined.

The elimination of dual diagnoses may have systematically biased present findings. In one study, the majority of participants were eliminated from analysis because it tended to diagnose personality dimensions and reactions simultaneously (Eisner and Cohen 1958). This may mean that participants with better prognoses were selected during analysis, leading to an overestimation of results compared to other studies that ascribed a single diagnosis to all participants. This would weaken the association between the consistency of the therapist's attitude and poor improvement rates.

#### 4.2.3 Comparing Improvement Rates

The translation of scores into a common rubric involved collapsing imprecise outcomes into their median, which may not be equal to the mean. The direction of the error is unknown, but it may be most prevalent in the three studies reporting binary outcomes (Eisner and Cohen 1958; Geert‐Jörgensen et al. 1964; Sandison and Whitelaw 1957b). Nevertheless, the true improvement rates of these low‐scoring studies are unlikely to reach as high as Martin (1967), with unimproved participants constituting 25%, 45%, or 50% of the included sample compared to Martin's 3%.

### 4.3 Future Directions

Cautious dosing was recommended in the present review. Today, the upper limit for standard doses has been estimated as 200 µg (Nichols and Grob 2018), and trials always exclude participants with a history of suicidal ideation or psychosis. These prudent measures might explain the absence of any suicides or cases of hallucinogen persisting disorder occurring in a review of 1072 LSD, psilocybin, and ayahuasca sessions (Romeo et al. 2024). The present findings also reinforce the consensus in current psychedelic research regarding the importance of preparation and a flexible therapeutic approach.

Other elements of psycholytic practice could also be introduced to psychedelic research in order to study their effects on an individual basis. Psychedelic treatment could be extended for those who fail to attain a mystical experience, and psychodynamic therapy could be introduced to gradually overcome the obstacles toward a psychodelic model (Yensen 1985). Additionally, low initial doses may support psychiatrists to offset dose‐response variability, although risk markers may be more subtle than therapeutic indications. Future research could investigate the effects of creative therapies during integration or of the presence of both male and female therapists to support anaclitic processes.

Alternatively, psychodynamic therapy may continue in its current form and be augmented by microdoses. In this framework, entheogens with a duration that aligns more closely with a conventional therapeutic session are highly applicable.

The recent possibility of developing custom artificial intelligence programs presents another way to research psycholytic therapy because they have the power and flexibility to analyze large amounts of disordered data. As of 2013, a single dose of an entheogen for research could cost as much as £1000 (Nutt et al. 2013), indicating that the 303 extant psycholytic studies (Passie 1997) are highly valuable. Psycholytic research could be a financially viable field in which to launch these research methods.

## 5 Conclusion

The large number of inaccessible studies underscores the neglect of psycholytic therapy and adds value to this realist review as it elucidates the protocols and procedures across more than 4640 sessions. Over 60 years since Sandison (1963, 36) declared that “much uncertainty still exists” in psycholytic therapy, further exploration remains due. The results suggest that when prudent methods are mobilized, psycholytic therapy could be safe and effective in severely ill patients.

## Author Contributions

**Hamish Grime**: conceptualization, writing – original draft, writing – review and editing, and methodology. **Eugenia Drini**: conceptualization, supervision, writing – review and editing. All authors: Approval of final manuscript.

## Funding

The authors have nothing to report.

## Data Availability

Data sharing is not applicable to this article as no new data were created or analyzed in this study.

## Appendix A Diagnostic Groupings Used for Adjustment of Improvement Rates

### A.1

**Table:**

Group title	Disorders in group
Depression	Depressive reaction; Depressive neurosis; Neurotic depression; Psychoneurotic depression; Endogenous mental depression; Tense depressive reactions in young; Tense depressive reactions in old.
Anxiety	Anxiety; Chronic anxiety; Anxiety state; Anxiety reaction; Anxiety neurosis; Primary anxiety neurosis; Anxiety neurosis with great mental tension; Chronic tension states; Phobic anxiety; Phobia; Phobic reaction.
Obsessive‐compulsive disorders	Obsessional; Obsessional neurosis; Classical obsessional neurosis; Obsessional reactions; Compulsive‐obsessive reation; Compulsive personality; Anancastic neurosis; Anorexia Nervosa; Hypochondriasis.
Hysteria	Schizophrenia; Schizophrenic reaction; Schizophrenic borderline; Residual schizophrenia; Paranoid‐schizophrenia; Paranoid; Sensitiver Beziehunswahn.
Psychotic disorders	Character neurosis; Narcissistic; Psychopathic; Psychopaths; Passive‐aggressive; Inadequate; Emotionally unstable; Infantile; Schizoid; Inadequate and schizoid.
Personality disorders	Character neurosis; Narcissistic; Psychopathic; Psychopaths; Passive‐aggressive; Inadequate; Emotionally unstable; Infantile; Schizoid; Inadequate and schizoid.
Excluded disorders	Cyclothymic; Addiction; Alcoholism; Sexual neurosis; Sexual disorders; Homosexuality; Balbutio; Mogigraphia; Paranoid‐obsessional; Older depressive patients with epilepsy; Anxiety neurosis associated with alcoholism, schizoid, or immature personality; Schizoid‐depressive; Paranoid‐obsessional; Self‐experiments by doctors; Impediment of speech

## Appendix B Translation of Raw Outcome Data to the Scoring System of Chandler and Hartman (1960)

### B.1

**Table:**

Score	Chandler and Hartman (1960)	Eisner and Cohen (1958)	Geert‐Jörgensen et al. 1964	Leuner (1963)	Martin (1957)	Martin (1967)	Sandison et al. (1954)	Sandison and Whitelaw (1957a)	Sandison and Whitelaw (1957b)	Whitaker (1964)
5	Outstanding	Improved	Improved	Recovered	Recovered	Recovered	Recovered	Recovered	Recovered or much improved	Recovered
4	Marked			Greatly improved	Greatly improved	Greatly improved	Greatly improved	Greatly improved		Much improved
3	Considerable									
2	Some (moderate)			Moderately or not improved	Slightly improved	Slightly improved	Moderately improved	Moderately improved		Improved
1	Slight improvement									Doubtful improvement
0	Little or no change	Unimproved	Unchanged		Not improved	Not improved	Not improved	Not improved	Not improved	Unchanged

## Appendix C Diagnosis‐adjusted Improvement Rates

### C.1

**Table:**

Diagnostic group	Chandler and Hartman (1960)	Eisner and Cohen (1958)	Geert‐Jörgensen et al. (1964)	Leuner (1963)	Martin (1957)	Martin (1967)	Sandison et al. (1954)	Sandison and Whitelaw (1957a)	Sandison and Whitelaw (1957b)	Whitaker (1964)
Depression	2.5	2.9	1.6	4.3			4.8	1.7	1.9	2.5
Anxiety	2.2		2.1	3.0	1.9	4.8	2.4	2.9		3.1
Obsessive‐compulsive disorders	1.8	3.0	1.0	3.0	2.2	3.4	3.5	2.5		2.6
Hysteria	3.5	2.6	1.6	2.7		4.1		2.5	1.8	
Psychotic disorders	2.0	2.4	1.8	4.2	4.6	3.5	3.8	1.6		3.4
Personality disorders	2.5	1.3		2.2		3.9	2.8	1.3		2.7
Mean	2.18	2.41	1.77	3.30	2.37	3.78	3.41	2.21	1.78	2.78

## Appendix D Clinically Relevant Ingredients in Psycholytic Therapy

### D.1

**Table:**

Author, year	Mean number of psycholytic sessions (n)	Upper limit of usual doses (µg)	Incremental dosing strategy (Y/N)	Prep sessions (Y/N)	Flexible or Consistent attitude of clinician (F/C)	Transference or Abreaction (T/A)	Clinician in constant attendance? (Y/N)	Pharmacological termination (Y/N)
Chandler and Hartman 1960	6.2	150	Y	Y	C	T	Y	Y
Eisner and Cohen 1958	4.6	150	Y	—	F	T	Y	N
Geert‐Jörgensen et al. 1964	—	400	Y	—	—	—	—	—
Leuner 1963	7	125	N	Y	F	A	N	N
Martin 1957	—	—	Y	—	—	A	N	Y
Martin 1967	20	—	N	Y	F	T	Y	N
Sandison et al. 1954	9	—	Y	Y	F	A	N	N
Sandison and Whitelaw, 1957a	10		Y	N	F	A	N	Y
Sandison and Whitelaw, 1957b	5	100	N	N	F	A	N	Y
Whitaker 1964	32.8	250	N	Y	F	A	Y	Y

## Appendix E Indications of Study Rigor

### E.1

**Table:**

Author, year	Participants included in analysis (N)	Possible outcomes for each participant (n)	Follow‐up time (months)	Participant severity (Low/ Medium/ High)	Outcome measure explained (✓/‐)	Dropout rate (%)
Chandler and Hartman 1960	78	6	—	M	**✓**	35
Eisner and Cohen 1958	13	2	6‐17	—	**✓**	24
Geert‐Jörgensen et al. 1964	114	2	12‐36	M	—	27
Leuner 1963	44	3	—	L	—	—
Martin 1957	47	4	—	—	—	—
Martin 1967	35	4	—	M	—	8
Sandison et al. 1954	21	4	—	H	—	4
Sandison and Whitelaw, 1957a	70	4	6	M	—	—
Sandison and Whitelaw, 1957b	14	2	—	H	—	0
Whitaker 1964	60	5	—	H	—	4
